# Single-plant GWAS coupled with bulk segregant analysis allows rapid identification and corroboration of plant-height candidate SNPs

**DOI:** 10.1186/s12870-019-2000-y

**Published:** 2019-10-08

**Authors:** Abiskar Gyawali, Vivek Shrestha, Katherine E. Guill, Sherry Flint-Garcia, Timothy M. Beissinger

**Affiliations:** 1 Division of Biological Sciences, University of Missouri, Columbia, USA; 2 USDA-ARS, Columbia, MO USA; 3 Division of Plant Sciences, University of Missouri, Columbia, USA; 4 Department of Crop Sciences, Georg-August Universität Göttingen, Göttingen, Germany; 5 Center for Integrated Breeding Research, Georg August Universität Göttingen, Göttingen, Germany

**Keywords:** Single plant genome wide association study, Maize, Plant height, FarmCPU, Bulk segregant analysis

## Abstract

**Background:**

Genome wide association studies (GWAS) are a powerful tool for identifying quantitative trait loci (QTL) and causal single nucleotide polymorphisms (SNPs)/genes associated with various important traits in crop species. Typically, GWAS in crops are performed using a panel of inbred lines, where multiple replicates of the same inbred are measured and the average phenotype is taken as the response variable. Here we describe and evaluate single plant GWAS (sp-GWAS) for performing a GWAS on individual plants, which does not require an association panel of inbreds. Instead sp-GWAS relies on the phenotypes and genotypes from individual plants sampled from a randomly mating population. Importantly, we demonstrate how sp-GWAS can be efficiently combined with a bulk segregant analysis (BSA) experiment to rapidly corroborate evidence for significant SNPs.

**Results:**

In this study we used the Shoepeg maize landrace, collected as an open pollinating variety from a farm in Southern Missouri in the 1960’s, to evaluate whether sp-GWAS coupled with BSA can efficiently and powerfully used to detect significant association of SNPs for plant height (PH). Plant were grown in 8 locations across two years and in total 768 individuals were genotyped and phenotyped for sp-GWAS. A total of 306 k polymorphic markers in 768 individuals evaluated via association analysis detected 25 significant SNPs (*P* ≤ 0.00001) for PH. The results from our single-plant GWAS were further validated by bulk segregant analysis (BSA) for PH. BSA sequencing was performed on the same population by selecting tall and short plants as separate bulks. This approach identified 37 genomic regions for plant height. Of the 25 significant SNPs from GWAS, the three most significant SNPs co-localize with regions identified by BSA.

**Conclusion:**

Overall, this study demonstrates that sp-GWAS coupled with BSA can be a useful tool for detecting significant SNPs and identifying candidate genes. This result is particularly useful for species/populations where association panels are not readily available.

**Electronic supplementary material:**

The online version of this article (10.1186/s12870-019-2000-y) contains supplementary material, which is available to authorized users.

## Background

Maize (*Zea mays.* L.) is one of the most widely grown crops worldwide because of its importance for food, feed, fuel, and raw material for industry [1]. In addition, it is also an important model species with tremendous phenotypic and molecular diversity. Molecular diversity is evident from different studies where millions of segregating markers have been observed, even using a modest population size [2–4]. Breeders have had remarkable success capturing this diversity to develop modern maize varieties that exhibited enhanced adaptation and production characteristics [5]. To continue developing improved varieties, the identification of genes or loci associated with important traits is the first among many steps required to leverage these genes for downstream use in breeding [6].

Plant height (PH) is an important agronomic trait in crop species such as maize. Breeders have identified a correlation between PH, grain yield, and biomass [7–9]. PH is a complex quantitative trait which has been explained by Fisher’s infinitesimal model, which posits that it is controlled by many genes with small effect [10, 11]. Also, PH is a highly heritable trait, although only a subset of the loci associated with PH have been identified [12–16]. Due to the agronomic importance of plant height, scientists have frequently studied it using conventional quantitative trait locus (QTL) mapping approaches [17–19]. QTL mapping has been proven to be a powerful approach to identify regions of the genome that contain the genes associated with important traits [20, 21]. For instance, several linkage mapping-based QTL studies have identified at least 5–12 loci associated with PH [17–19]. Collectively, Gramene shows more than 219 QTLs identified for PH in maize across an assortment of mapping populations (http://archive.gramene.org/qtl/). Many of the previous studies on PH have identified gibberellin (GA) and brassinosteroids (BR) as major hormones involving in stem elongation [22–25]. In addition, auxin biosynthesis and signaling also play a key role in regulating stem length [26]. However, the QTL mapping approach has limitations, the first of which is the fact that it requires the creation of a mapping population, which can be a slow and resource intensive process. Also, mapping resolution is typically low, often encompassing several centimorgans including several hundred genes. Another limitation is that QTL mapping captures only small portion of the phenotypic variation of many agronomic traits—that which differentiates the two parents that are crossed to form a mapping population [27, 28].

Modern high throughput genotyping techniques have made the identification of single nucleotide polymorphisms (SNPs) much easier [29]. SNP markers are often used to conduct genome wide association studies (GWAS) to identify genes associated with the variation in the quantitative traits including many physiological, molecular and cellular traits [30]. GWAS identify associations by exploiting the genetic diversity within a species that contributes to the phenotype. Historical recombination events captured in the population greatly increase mapping resolution. However, most GWAS in crops have previously been performed using populations consisting of panels of inbred lines phenotyped in multiple replications [31–34]. In contrast, a new approach, F-one association mapping (FOAM), was used to perform GWAS with 4417 maize landrace accessions leveraging heterozygous loci. The original FOAM method involved a reproduction step during which each landrace accession was crossed to a small number of single cross hybrid females, and phenotyping was done on each family as a replicated set of progeny [35]. Unreplicated phenotyping of individuals is common in human and animal GWAS, where replicating genetically-identical individuals can be difficult or impossible [36, 37]. The ability to conduct replicated experiments in order to reduce measurement error is possible and relatively straight-forward in in self-compatible plants. Because of this, the use of individual-plant phenotypes is not a standard practice in crop plants. But, if individual-plant phenotypes can be used for GWAS in plants, this has the potential to drastically reduce the time and resources required to complete an experiment.

Bulk segregant analysis (BSA) is an alternative approach that utilizes genome-wide marker data to identify the casual genes for complex traits [38]. BSA in plants was initially used to detect markers in a segregating population to identify disease resistant genes [39]. In [33], DNA libraries were constructed using bulks of pooled F2 samples of phenotypically extreme progeny that were generated from a cross of the two phenotypically contrasting parents. Then, markers were screened for DNA variants with significantly different frequencies between the pools. BSA has already proven to be useful technique in crop species to detect QTL of large effect such as resistance to abiotic/biotic stress or to map qualitative mutants [40–42]. Analogously to earlier BSA studies that involved bi-parental or other structured populations, modified implementations of BSA can be performed on unstructured populations by leveraging sequence data. Such an approach was previously implemented in maize by [43], where it was called xp-GWAS.

Here, we perform a GWAS using a maize landrace known as Shoepeg, which is an unimproved population of randomly-mated individuals adapted to an environment and which possess particular morphological attributes that are characteristic of that landrace. As segregation is a fundamental pre-requisite for any mapping study, the shoepeg landrace ideally contains segregating variation throughout the genome because of the fact that the landraces are created through random mating and usually tend to be heterogenous. Therefore, at any locus many individuals may be homozygous or heterozygous. We focused this study on plant height, which serves as a model for moderately complex traits with the ultimate goal of applying this method to more difficult or expensive phenotypes. We implement our GWAS on single-plant genotypes and phenotypes, and therefore refer to the approach as single-plant GWAS (sp-GWAS), since individual segregating plants are genotyped and phenotyped for the association analysis. As we show, an important benefit of sp-GWAS is that it can be efficiently combined with BSA for rapid and independent corroboration of candidate SNPs .

Herein, we describe the application of this sp-GWAS pipeline to PH as a model-trait. We demonstrate that with inexpensive genotyping, a moderate number of genotyped and phenotyped individuals, and a moderate to high-heritability trait: PH, our pipeline involving sp-GWAS and BSA-based SNP corroboration, can be used to successfully and efficiently identify candidate loci. Loci identified by our pipeline include previously identified candidate genes, which are further validated by performing BSA using extreme phenotypes on same population.

## Results

### GWAS and BSA PIPELINE OVERVIEW

**Fig. 1:**
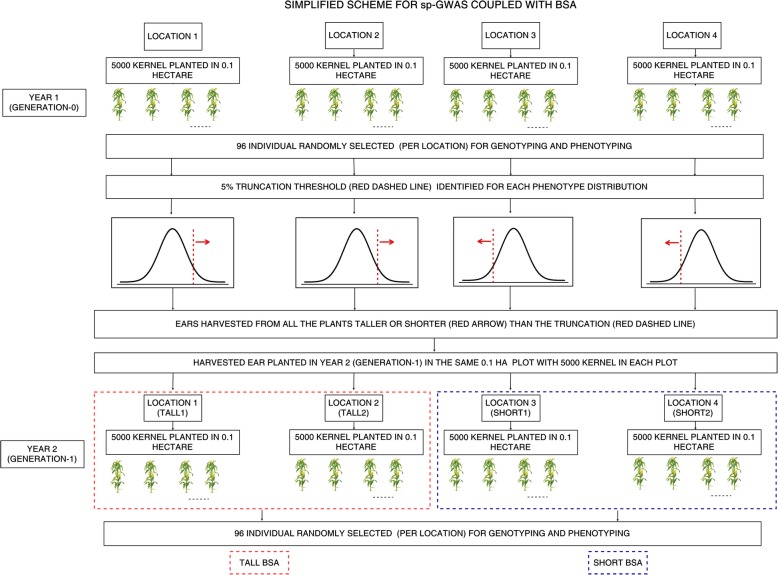
Schematic pipeline of sp-GWAS coupled with BSA. Year1 (Generation-0): 5000 plants were planted in ~ 0.1 ha plots in four locations (20,000 plants total) and 96 individual plants were selected randomly in each location (384 in total) for genotyping and phenotyping. Based on the phenotypic distribution of 96 plants, the ~ 5% truncation threshold was identified for each location. All the ears from plants taller (Location 1 and 2) or shorter (Location 3 and 4) than the truncation threshold were harvested. Year2 (Generation-1): Harvested seeds (5000 kernels) from year1 (Generation-0) were grown again in same location and 96 plants per location (384 in total) were genotyped and phenotyped in the same manner as in year1. These populations are now named based on the selection regime; Generation1-Tall1, Generation1-Tall2, Generation1-Short1 and Generation1-Short2. Association analysis was done using all 768 (384 × 2) phenotyped and genotyped plants. Offspring of the selected individuals from year1 were used for the modified bulk segregant analysis using tall and short populations to define in silico bulks

Details describing our pipeline to efficiently combine sp-GWAS with BSA for rapid identification and corroboration of candidate trait-associated SNPs are described in detail in the methods section of this manuscript. Therefore, we have included only an overview of the approach here, as well as a summary figure to demonstrate our pipeline (Fig. 1). In Generation-0, we planted 5000 plants from the Shoepeg population in each of four separate 0.1-ha plots (20,000 plants in total). In each plot, 96 individual plants (384 in total) were phenotyped for plant height and genotyped using GBS [44]. From the phenotypic distribution of these plants, ~ 5% truncation thresholds were identified for each of the 0.1 ha plots, and ears from plants taller (2 plots) or shorter (2 plots) than the truncation thresholds were harvested. In Generation-1, seeds from the harvested ears were again grown in four 0.1 ha plots with 5000 plants in each, and 96 plants/plot were genotyped and phenotyped (384 in total). All 768 (384 × 2) phenotyped and genotyped plants were used for sp-GWAS, and allele frequencies computed from the 96-plants/plot in Generation-1 were used to indicate allele frequencies of phenotypically extreme Generation-0 plants for BSA. Scripts to implement our pipeline and analysis are available online (https://github.com/abi01/sp-GWAS).

### Phenotypic evaluation

**Fig. 2:**
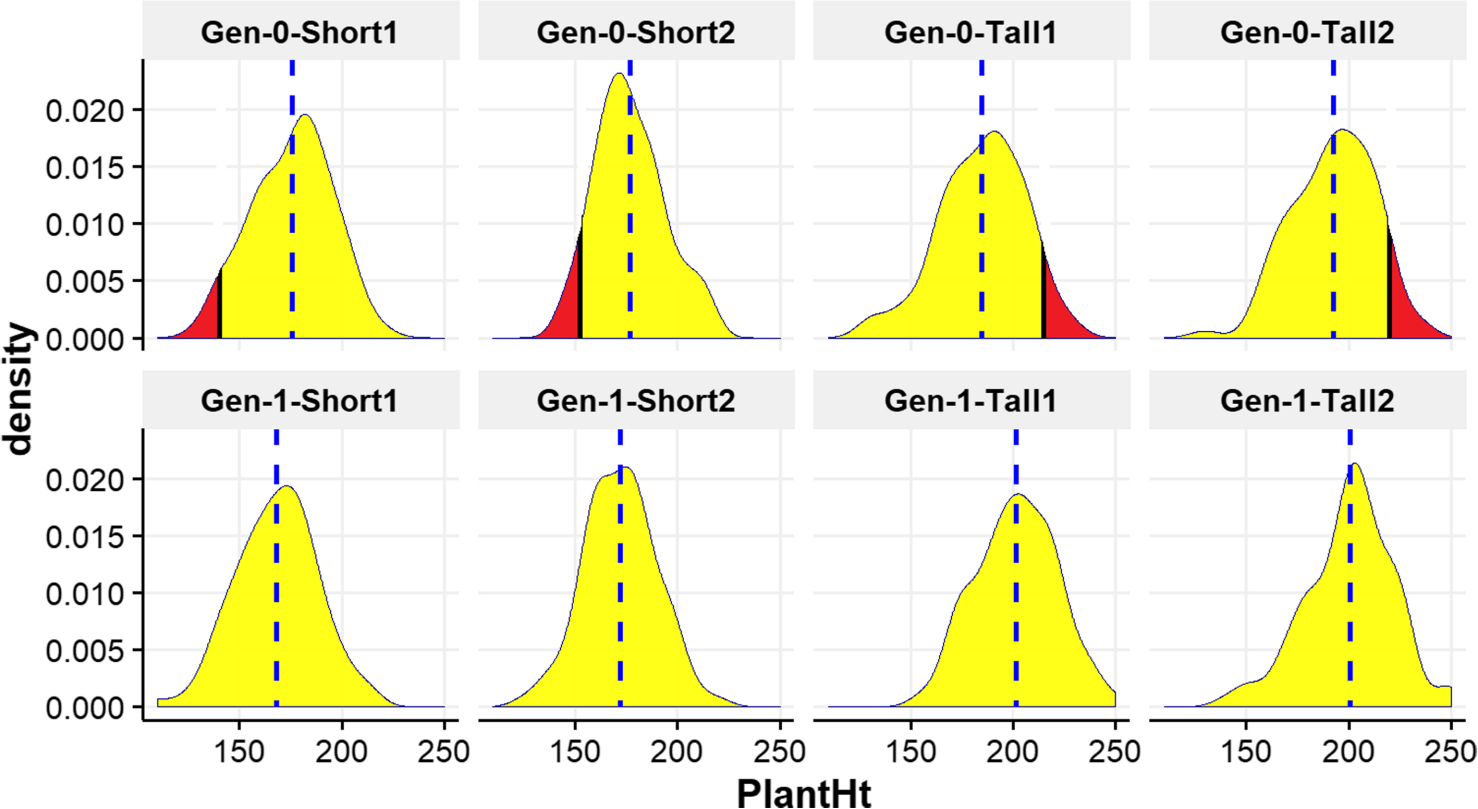
Phenotype distribution of plant height (PH). The density plot shows the phenotypic distribution of plant height in all four locations for two generation (top row: Generation-0 and bottom row: Generation-1). The blue dashed line shows the average value of each distribution. The red portion of the Generation-0 distribution represents plants selected to form Generation-1

We measured PH for 768 individuals across two Generations and four locations: 384 from Generation-0 in 2016 and 384 from Generation-1 in 2017. Descriptive statistics for PH across all environments and both generations are provided in Table 1. The phenotypic distribution of Shoepeg PH in all four locations in both generations shows wide variation and an approximately normal distribution (Fig. 2). Average heritability was computed using GCTA (*h*^*2*^_*GCTA*_) for single-plant PH across all four locations in both generations was estimated to be 0.7463, which indicated that the major proportion of phenotypic variation detected in PH is due to genetic factors making it suitable for association analysis. Realized heritability was also computed using the breeder’s equation (*h*^*2*^_*bs*_) [45]. As described in more detail in (Additional file 1), environmental differences with respect to selection environments in different locations and years complicate our application of the breeder’s equation to estimate heritability in this setting. Even so, using this technique we conservatively estimated an average *h*^*2*^_*bs*_ of 0.31 for plant height (Additional file 1). We are more confident in our *h*^*2*^_*GCTA*_ estimate than our *h*^*2*^_*bs*_ estimate of heritability, although both show a relationship between genotype and phenotype that can be leveraged for mapping. Other researchers have successfully implemented GWAS in animal populations with similar heritabilities and sample sizes [46, 47].

**Table 1:** Descriptive statistics for field trials, and plant heights observed for Cycle-0 and Cycle-1 plants

	Gen-0-Tall1	Gen-0-Tall2	Gen-0-Short1	Gen-0-Short2	Gen-1-Tall1	Gen-1-Tall2	Gen-1-Short1	Gen-1-Short2
Year	2016	2016	2016	2016	2017	2017	2017	2017
Field Location	Rollins	Rollins	Genetics	Genetics	Vineyard Low	Rollins	Vineyard High	Genetics
Number	96	96	96	96	96	96	96	96
Minimum (cm)	130	130	145	130	155	140	110	125
Maximum (cm)	230	235	215	220	250	250	215	220
Range (cm)	100	105	70	90	95	110	105	95
Median (cm)	190	195	175	180	200	200	170	175
Average (cm)	184.8	192.5	176.8	175.6	201.5	200.5	168.1	172.1
Std. Deviation (cm)	20.9	19.9	16.9	19.3	19.6	21.3	19.8	17.6
Selection Truncation Threshold	> 215 cm	> 220 cm	< 140 cm	< 152 cm

### Genome wide association analysis

**Fig. 3:**
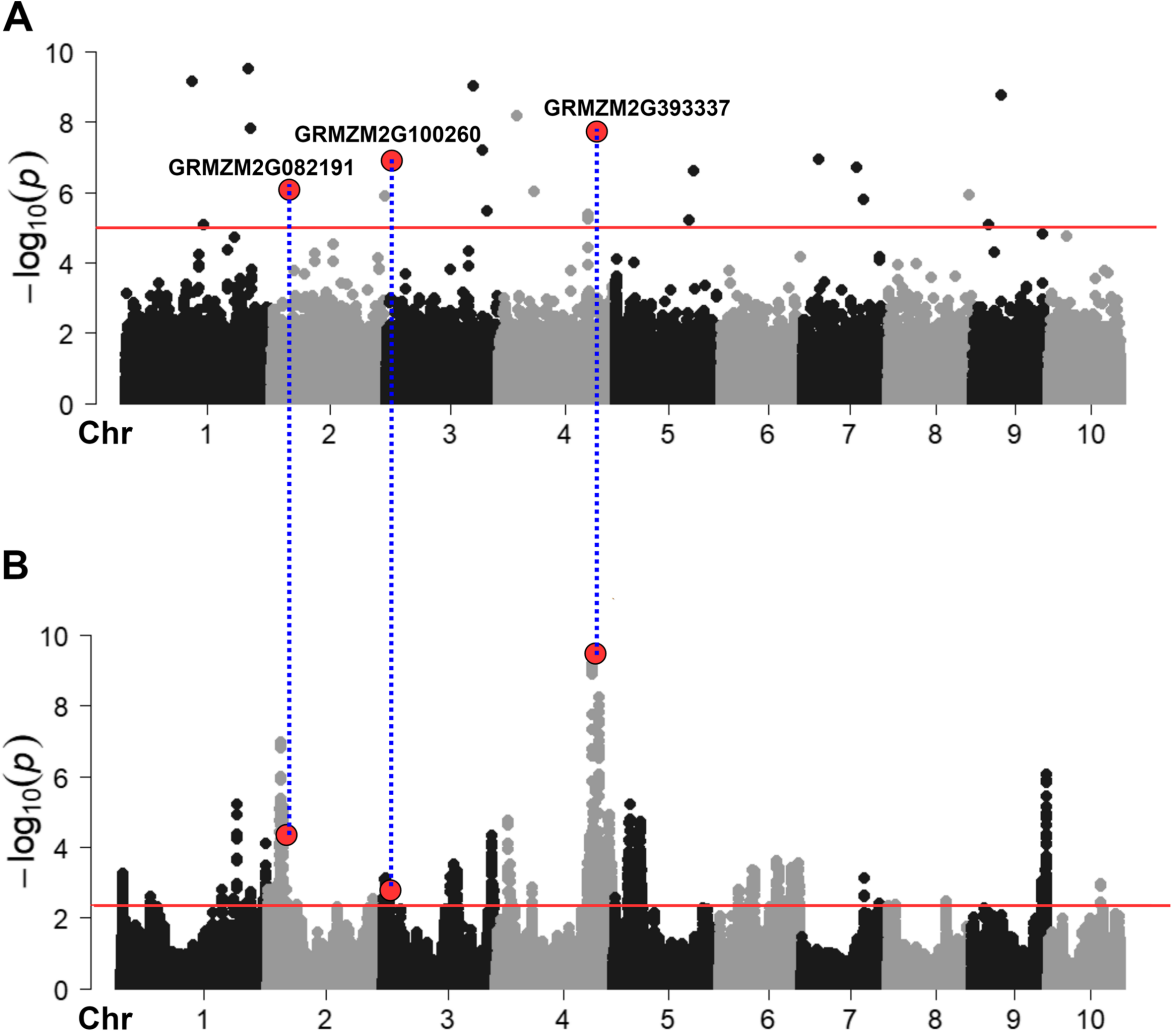
Genome wide association mapping of plant height. **a** Manhattan plot of the single plant genome-wide association analysis (sp-GWAS) using FarmCPU. GWAS identified total of 25 significant SNPs for plant height that surpassed the significance threshold (*P* ≤ 0.00001). **b** Manhattan plot of the bulk segregant analysis (BSA) sequencing method for mapping plant height. BSA identified 37 significant regions (0.5% outlier). Red horizontal lines denote the significance threshold both for sp-GWAS and BSA. The overlapping SNPs in both GWAS and BSA is highlighted in red dots and the gene containing those three SNPs are highlighted and are aligned by blue dashed line

Principal component analysis (PCA) did not reveal substantial population structure within the overall Shoepeg population or across Generations (0 or 1) or selection regimes (tall or short) after normalization (Additional file 2). This was expected since Shoepeg is a single random-mating population and therefore should not contain major admixture features or reflect several generations of drift genetically separating plants. Therefore, we used only generation (cycle 0 and cycle 1) and selection regime (tall and short) as covariates in our GWAS model. GWAS was performed using FarmCPU. A total of 25 significant SNPs associated with plant height (*P* ≤ 0.00001) were detected by GWAS (Fig. 3a, Additional file 3, Table 2). This is low compared to some association studies for PH that have been previously conducted in maize [12, 48] likely due to the restricted genetic diversity of the Shoepeg population as compared to broad diversity panels. These 25 significant SNPs explained 48 and 36% variance in Gen0 and Gen1 respectively. The two most significant SNPs were found on chromosome 1 with *P* values 3.15e-10 and 7.17e-10, respectively. The effect size of significant SNPs varied from − 5.77 to 6.47 cm, with mean effect size of 0.63 cm.

**Table 2:** Top QTN associated with plant height identified by the sp-GWAS method

Chr	Position	*P*-value	Maize Gene Annotation	Nearby candidate Gene
1	136,947,083	7.17E-10		
1	160,450,782	8.35E-06	GRMZM2G081151: Camelliol C synthase	
1	253,355,509	3.15E-10	GRMZM2G066234: Protein OSB2 chloroplastic	GRMZM2G366373: Auxin-responsive protein IAA4; Ortholog of indole acetic acid 3/short hypocotyl 2 in *A. thaliana*
1	258,740,929	1.53E-08		
2^a^	31,215,734	7.03E-07	**GRMZM2G082191:** Probable leucine-rich repeat receptor-like protein kinase	**GRMZM2G082191 (SNP in candidate):** receptor like protein kinase; Brassinosteroid insensitive function in rice
2	236,038,975	1.27E-06		
3^a^	9,953,933	1.01E-07	GRMZM2G100260: D-Tyr-tRNA (Tyr) deacylase family protein	GRMZM2G004696: Aux/IAA transcription factor 8
3	179,174,157	9.31E-10	GRMZM2G400390: Laccase-7	GRMZM2G449033: nana plant1
3	198,776,203	6.46E-08		
3	208,434,550	3.43E-06		
4	36,939,527	6.59E-09	GRMZM2G508530: unknown function	GRMZM2G170232: tcp- transcription factor 39; Ortholog of tcp transcription factor 1 in *A. thaliana*
4	74,075,452	9.21E-07		
4	184,329,977	5.72E-06		
4	184,329,983	5.72E-06		
4	184,329,984	5.28E-06		
4	184,329,985	4.29E-06		
4^a^	199,976,446	1.38E-08	**GRMZM2G393337:** 3-hydroxy-3-methylglutaryl-coenzyme A reductase 2	**GRMZM2G393337 (SNP in candidate);** Ortholog of Hydroxy Methylglutaryl CoA Reductase 1 (hmg1) in *A. thaliana*
5	152,583,112	6.12E-06		GRMZM2G161299: mcf1 - mitochondrial carrier family protein1
5	161,902,893	2.37E-07	GRMZM2G123587: Protein ROS1	
7	34,193,779	1.15E-07	GRMZM2G092129: Calcium-dependent lipid-binding (CaLB domain) family protein	
7	111,286,404	1.91E-07		
7	125,687,609	1.65E-06		
8	166,967,198	1.15E-06		
9	32,107,065	8.33E-06		
9	59,694,089	1.67E-09		

^a^SNPs/regions that overlap between sp-GWAS and BSA

### Bulk segregant analysis

BSA served as a valuable validation add-on to our sp-GWAS pipeline that provided corroboration of the most promising GWAS SNPs at minimal additional cost. (Fig. 1). BSA in this study was computed by selecting top 5% as tall PH bulks and bottom 5% as short PH bulks from Gen-0. A total of 243,303 SNPs were compared for allele frequency differences between the 192 individuals genotyped in Cycle 1, which represented the tallest and shortest individuals in Cycle 0. Allele frequency was estimated based on an *in-silico* bulk of the individuals (details in methods). A total of 1201 significant markers across 37 regions were identified. Significant BSA SNPs with frequency of 0.05 or less was ~ 2% of the total significant SNPs. The top two significant regions were found on Chromosomes 2 and 4, and these encompassed 15.7 and 28.3 Mb, respectively (Table 3; Fig. 3b).

**Table 3:** Significant genomic regions and most significant SNP in each region identified by bulk segregant analysis (BSA)

Chr	Left Position	Right Position	Length (kb)	Most significant SNP (within the region)	*P*-value of most significant SNP
1	3,882,962	5,339,260	1456.3	5,330,693	5.47E-04
1	60,757,940	62,234,211	1476.3	60,808,467	2.54E-03
1	208,068,095	208,216,068	148.0	208,145,209	1.67E-03
1	230,758,325	230,895,709	137.4	230,796,501	3.04E-03
1	238,020,521	238,027,732	7.2	238,024,306	5.99E-06
1	258,497,186	258,507,663	10.5	258,502,438	3.89E-03
1	267,066,207	267,228,747	162.5	267,219,432	1.82E-03
1	293,218,485	299,645,056	6426.6	299,541,586	8.15E-05
2	2,130,342	2,227,154	96.8	2,208,251	1.61E-03
2^a^	17,397,531	33,383,795	15,986.3	27,507,105	1.10E-07
2	41,034,083	41,067,877	33.8	41,038,235	1.57E-03
2	217,455,023	217,555,483	100.5	217,491,376	3.01E-03
3^a^	2,679,766	12,559,796	9880.0	6,936,331	7.33E-04
3	139,209,369	139,253,807	44.4	139,252,674	6.97E-04
3	146,347,706	154,225,475	7877.8	146,397,740	3.02E-04
3	223,622,887	229,075,692	5452.8	225,291,181	4.88E-05
4	26,431,986	26,532,076	100.1	26,434,686	1.79E-05
4	31,498,513	32,679,484	1181.0	31,546,290	2.97E-04
4	76,411,353	76,423,428	12.1	76,411,660	1.34E-03
4^a^	188,388,447	216,889,443	28,501.0	198,947,559	3.78E-10
4	229,854,776	239,419,902	9565.1	235,360,468	1.20E-05
5	3,996,779	4,035,497	38.7	39,912,899	7.54E-04
5	31,366,406	34,883,247	3516.8	34,774,969	6.08E-06
5	39,796,417	39,929,581	133.2	39,912,899	7.55E-04
5	49,927,102	58,754,489	8827.4	56,940,744	1.98E-05
6	39,702,906	39,826,253	123.3	39,724,316	1.62E-03
6	63,924,853	68,656,185	4731.3	68,361,841	4.57E-04
6	74,549,141	74,625,826	76.7	74,625,635	5.00E-04
6	117,970,384	124,481,894	6511.5	118,935,475	2.39E-04
6	135,039,689	135,267,591	227.9	135,110,742	3.49E-03
6	147,215,787	151,134,287	3918.5	151,039,462	3.86E-04
6	158,676,084	166,187,944	7511.9	164,751,856	2.96E-04
7	130,739,179	130,913,149	174.0	130,910,813	7.33E-04
7	159,797,611	159,817,662	20.1	159,809,085	4.05E-03
8	121,946,436	121,954,911	8.5	121,951,520	3.46E-03
9	144,576,519	153,778,981	9202.5	152,798,129	8.53E-07
10	108,394,057	108,436,541	42.5	108,399,093	1.08E-03

^a^SNPs/regions that overlap between sp-GWAS and BSA

### Candidate gene identification

Based on the information available from the B73 reference genome *v*3 [49], 9 of the 25 GWAS-identified SNPs are located within gene models. Of these, four are located in translated regions and the remaining five are in introns. Based on gene annotation information available in MaizeSequence (http://ensembl.gramene.org/Zea_mays/Info/Index) and MaizeGDB (http://www.maizegdb.org/gbrowse), we further evaluated the potential function of candidate genes located near significant loci. Fourteen annotated gene candidates were located within 150 kb of the 25 significant SNPs, and among these nine have unknown function.

We identified several promising candidate genes based on orthology with *Arabidopsis thaliana* genes involved in plant stature*.* Maize *TCP-transcription factor39*, GRMZM2G170232, which is located 29 kb downstream of a significant SNP on chromosome 4 (position 36,939,527), is an ortholog of *tcp-transcription-factor1 (tcp1)* of *A. thaliana*. Another significant SNP on chromosome 4 is located within GRMZM2G393337, which is orthologous to *hydroxy methylglutaryl coa reductase 1 (hmg1/hmgr1)* of *A. thaliana* which causes dwarfing when mutated due to suppression of cell elongation [50]. Interestingly, the SNP in GRMZM2G393337 had the largest effect of 6.4 cm. We identified a gene GRMZM2G366373, which is an ortholog of *A. thaliana iaa3 - aux/iaa-transcription factor 3 (iaa3)/short hypocotyl 2 (shy2),* located 6.5 kb downstream of the peak SNP on chromosome 1 (GRMZM2G066234; *P* = 3.15e-10). Gain of function *shy2* mutants shows dwarf phenotype in *A. thaliana* [51]*.* A highly significant SNP on the long arm of chromosome 3 (position 179,174,157) is 133 kb upstream of *nana plant 1 (na1)* which causes dwarfing when mutated in maize and is homologous to the *de-etiolated2 (det2)* gene involved in brassinosteroid synthesis in *A. thaliana* [52]. We identified *mcf1 - mitochondrial carrier family protein1* as a candidate gene located 112 kb upstream of a significant SNP on chromosome 5 (position 152,583,112). This is the same class of family protein was identified as a candidate gene for PH in [31].

Additional potential candidate genes associated with PH were identified from BSA. In total, BSA identified 37 regions distributed across all 10 chromosomes. Since many of the BSA regions were relatively large (mean size 3.5 Mb), there is a strong possibility that some of the candidates within these regions are not causal in this experiment. Nevertheless, genes candidate genes within the BSA regions included maize *arftf2 – auxin response factor transcription factor 2,* located within 16 kb in chromosome 1, which is orthologous to the putatively expressed *OsARF18.* Rice transgenic plants with *OsARF18* alleles are short in height compared to wild type [53]. We also identified *nana plant2* (*na2*), the maize ortholog of the *A. thaliana DWF1* gene, on chromosome 6. *DWF1* plants exhibit severe dwarfism similar to BR- deficient mutants. Several GRAS-population transcription factors involved in gibberellic acid signaling were identified in the BSA: *Gras45* (GRMZM2G02809) and *gras69* (GRMZM2G153333) are identified in within the significant BSA regions in chromosome 9 and chromosome 6 respectively. In previous research, *gras45* was identified as a significant GWAS hit in tropical lines [48].

### Overlapping GWAS hits with BSA regions

BSA identified 37 regions and GWAS identified 25 significant SNPs associated with PH. Three significant GWAS SNPs overlapped with BSA regions: GRMZM2G082191 on chromosome 2 (position 17.4–33.2 Mb), GRMZM2G100260 on chromosome 3 (position 2.6–12.5 Mb), and GRMZM2G393337 on chromosome 4 (position 188.4–216.8 Mb). The candidate for the chromosome 4 region is the ortholog to *hydroxy methylglutaryl coa reductase 1 (hmgr1)* in Arabidopsis as discussed above. A second overlapping SNP/region is located on chromosome 2 in GRMZM2G082191, a receptor like protein kinase, orthologous to rice (LOC_Os04g42700.1) and Arabidopsis (AT5G63930.1). The third overlapping SNP/region located on chromosome 3 within GRMZM2G100260 was related to D-Tyr-tRNA (Tyr) deacylase family protein. None of these genes have functions obviously related to PH based on their gene annotations per se. However, two more likely candidate genes are located near GRMZM2G100260 and still within the BSA region on chromosome 3: *dwarf plant1 (d1;* GRMZM2G036340*)* was identified ~ 500 Kb away from GRMZM2G100260; and *iaa8 - aux/iaa-transcription factor 8 (iaa8;* GRMZM2G004696*),* a homolog to Arabidopsis (axr3/ iaa17) was located 122 kb upstream of GRMZM2G100260.

## Discussion

Genome wide association studies have been extensively used to identify candidate genes associated with complex traits [54]. Plant height is a commonly studied complex trait because it is a relatively simple phenotype to measure and because of its relationship with biomass [55], lodging resistance [56], and grain yield [57]. Association studies for maize plant height have been conducted using a variety of populations and marker sets [12–15, 31, 48, 58]. GWAS in plant genetics has been very successful for identifying causal genes for complex quantitative traits such as plant height, vegetative architecture, reproductive architecture and metabolic processes [30, 59]. Like GWAS, BSA is a technique to identify markers associated with a phenotype. The development of next generation sequencing has made the BSA approach much more feasible for mapping casual genes [60]. Initially BSA was used to analyze model organisms such as Arabidopsis and yeast [38, 61]. More recently this approach has been used in important crop species including rice [62, 63], soybean [64, 65], and maize [66–68]. All of these studies successfully identified significant QTL and candidate genes associated with traits.

Conventional GWAS is used to identify casual SNPs associated with important traits in crop species. However, almost every plant GWAS leverages a panel of inbred lines [30]. Recently an approach called FOAM was introduced, which involves the use of non-inbred landraces evaluated in un-replicated trials [35]. However, this approach still requires making a test cross to evaluate the phenotype for the association mapping. Using inbreds can increase the length and expense of a study if inbreds are not available beforehand, and because each inbred line must be planted separately (e.g. in its own row/plot) to maintain its identity. A recent association study to identify regions associated with kernel row number used pooled sequencing of individuals from a previously-studied diversity panel [43]. Although this approach cuts down the genotyping expense, it still requires generation of a mapping population and large phenotypic trials. In contrast, sp-GWAS relies on the use of individual-plant phenotypes scored within a single heterogeneous, random-mated population. GWAS on single-individuals is commonplace outside the plant world—for human [69, 70] and animal [71–73] GWAS, single-individual phenotypes have very successfully been used for mapping, as inbred panels are rarely available or impossible to create. Still, to ensure that sp-GWAS results are valid, the pipeline implemented in this study additionally allows the efficient combination of both the GWAS with BSA for corroboration of results (Fig. 1).

The importance of plant height for plant genetic studies has been recognized since Mendel [74]. Much research has been conducted trying to elucidate the molecular mechanisms explaining the wide variation observed for PH. Based on our analysis of the Shoepeg maize population using sp-GWAS and BSA, we identified a collection of major known candidate genes for PH in maize. However, only a limited number of additional putatively PH-related SNPs were identified by our study. A potential reason for this is that our study was only capable of identifying causal variants that are segregating in the Shoepeg population.

Many previous association studies for plant height and reverse genetics approaches using dwarf mutants have identified loci that are involved either in BR and GA synthesis or signaling. Both of these hormones have shown a direct impact on plant height or shoot length [23, 75]. M Suzuki*,* et al. [50] demonstrated that *hmg1* mutants show a similar phenotype to those of BR deficient mutants where the cell elongation is suppressed resulting in a dwarf phenotype. A recent publication identified PH QTN using GWAS in a panel of exotic introgression lines in the Stiff Stalk and Non-Stiff Stalk backgrounds [76]. Our study identified a significant overlapping SNP (both sp-GWAS and BSA) on chromosome 2 within the genic region of GRMZM2G082191 which was identified as a candidate gene by Hu et al. [76]. GRMZM2G082191 encodes a receptor like protein kinase and has a putative brassinosteroid insensitive function in rice [76]. Another study by [15] used joint linkage QTL mapping and joint linkage GWAS to identify the PH associated QTL and QTNs in the US-NAM and the North Central Region Plant Introduction Station (NCRPIS) Ames diversity panel. We identified *d1* as a major QTN in our study (both GWAS and BSA) which coincides with the major QTN identified in maize NAM populations [15]. *D1* encodes ZmGA3ox which catalyzes the GA biosynthesis in maize and its mutant shows phenotype of dwarf PH [77–79]. *Na1* is another important gene in BR synthesis and affects PH [80]. It was identified as one of the candidate genes in the QTL study of PH using recombinant inbred lines [81]. In our study, na1 was identified only in the sp-GWAS but not in the BSA.

Importantly, our pipeline demonstrates that with a very limited amount of additional labor, BSA can be combined with sp-GWAS for independent candidate SNP corroboration. Our GWAS was conducted across two years and four locations of observation, and by including an additional screening and selection step at the end of the first year, we were able to include BSA without even conducting additional sequencing. It is worth noting that in the case of PH, this additional screening step could be achieved in a very short time by walking through each field with measuring sticks (0.5–1 h for a year-location with a crew of four people). For a single year-location, 5000 k seeds were planted in 0.1-ha area. Plants were randomly selected, and phenotyping and genotyping was done on those randomly selected individuals for both the year. However, the difference is that in the first year, divergent selection was conducted based on the top or bottom ~ 5% of individuals as tall and short PH bulks. This approach allowed us to use genotypic and phenotypic data from both years for the association analysis, while only genotypic data from the second year was used for BSA. No spatial checks were incorporated in our experimental design in order to prevent pollen-contamination that would have been problematic for our BSA results. However, the incorporation of checks in future study may represent a promising way to confirm field uniformity, especially if a trait other than PH Is being assessed so that plants can be de-tasseled without the phenotype being affected.

Our study also demonstrates that significant associations can be achieved using sp-GWAS in a heterogeneous, random-mated population, such as an open pollinated maize landrace. Moreover, we were able to obtain corroborating evidence for a subset of the identified SNPs using BSA, which also provided an additional collection of putative QTL for PH. As was shown in a simulation study by Dell’Acqua, et al. [16], for a trait with 70% heritability, at least 500 individuals are needed to detect associations between markers and the trait. Field studies also show that an increase in number of individuals improves the power to detect marker-trait association [82, 83]. AD LongCH Langley [47] demonstrated that the power of association between marker and trait depends on the variation attributable to quantitative trait nucleotide (QTN) and the number of individuals. In our association study, we used 768 individuals with 306,522 SNPs (MAF < 0.05) to identify 25 significant SNPs (*P* ≤ 0.00001) associated with PH. While 25 associations is not tremendous based on a comparison to other PH experiments (references), a potential reason for this discrepancy, in addition to experimental power considerations, is that Shoepeg is a single populations with limited genetic variation.

As an add-on to the sp-GWAS pipeline, BSA was used to identify loci associated with PH by selecting divergent phenotypes from Generation-0. Using BSA on the population, we identified 37 genomic regions for PH. We identified a larger number of QTL in BSA than in GWAS. This was expected based on simulations that have shown that BSA has increased power to identify minor and rare alleles even of very small effect [38, 84]. Of the 37 QTL mapped for PH, three significant GWAS associations fall within distinct BSA peaks on chromosomes 2, 3 and 4, while other BSA peaks are located near significant SNPs (Tables 2 and 3).

In this study we demonstrated that sp-GWAS can efficiently and affordably produce results comparable to those from conventional GWAS experiments. Many of the candidate gene identified from the sp-GWAS are the major quantitative genes controlling the plant height. In-spite of the fact that we looked at one maize landrace population with limited genetic variation, we still successfully identified many candidate genes that have been implicated in standard GWAS studies. The corroboration of results from our linked but independents BSA for three of these SNPs provides additional evidence that our implementation of sp-GWAS is effective. Most of the previous validation work in conventional GWAS has been done using linkage mapping, and BSA has generally been used to validate either linkage mapping or pooled GWAS [43, 85]. However, BSA has been proven effective for mapping candidate QTLs [43, 69, 86–88].

There are several potential factors contributing to less number of overlapping signals identified by sp-GWAS and BSA. First of all, single plant measurements have an inherently lower heritability than plot-based phenotypes, and this certainly lowers the power of our approach. Also, BSA resolution is heavily dependent on the recent recombination pattern from one study-generation whereas association study is based on the ancient history of recombination. Finally, the power of identifying candidate gene in BSA depends on the tail size (number of individuals in the bulk) [86]. However, for the three regions that did overlap, our pipeline combining sp-GWAS and BSA provides strong evidence of a causal association. In this study BSA was done in 384 individuals (192 in each bulk only from generation 1) compared to GWAS which was done in 768 individuals.

Due to macro- and micro-scale variation between plants measured in field settings, researchers are often hesitant to utilize single-plant measurements. Instead, it is common to proceed by averaging measured values across a plot. Our results demonstrate that this practice may not always be necessary, particularly given the fact that plot-based experiments take up substantially more space, time, and effort than single-plant measurements. In our case, planting, phenotyping and harvesting was achieved in approximately 1 h. for each year-location with a crew of four people. It is worth noting that conducting studies based on a plot-design introduces alley-effects [89], which are not present in a single-plant experiment such as that described herein. However, our design may be further improved by the incorporation of appropriate checks and spatial variation into our model. This approach may be particularly beneficial in crops where association panels are unavailable or in which inbreeding is not feasible.

In a practical breeding setting, direct phenotypic selection for PH is likely more efficient than utilizing QTL in marker-assisted selection scheme. We are therefore using PH as a model for traits with moderate genetic complexity, but which may be more labor intensive or expensive to evaluate. Depending on the goals of the breeding program, PH could be targeted as part of a multiple-trait index along with other traits using genomic selection. Results from association mapping in a single landrace population, as implemented here, instead of in a more diverse panel, may be useful for incorporating genetic variation from a specific donor population into elite breeding material. Also, identification of significant loci in one setting can have discovery implications for identifying or generating new variation at genes of interest in other populations. Even with these advances, the gap between identifying and incorporating QTLs from GWAS into marker assisted selection pipelines for trait under improvement is unlikely to be affected.

## Conclusion

In conclusion, herein we have demonstrated a pipeline whereby sp-GWAS be powerfully coupled with BSA to efficiently identify significant trait-associated SNPs. The major advantage of using this approach is its simplicity, time-requirement (on the field and off field), and low cost. Our approach we described can be compared with the concept of FOAM [35], in which where multiple landrace populations are studied. The similarity between both approaches is that they both use heterozygous individuals, but differences include that FOAM involves sampling a large number of very diverse landraces and phenotyping multiple individuals for replication at the family-level, while sp-GWAS involved phenotyping completely unreplicated individuals. This means that the cost of sp-GWAS is extremely low, even after it is coupled with BSA to achieve immediate independent corroboration of results. However, the power of sp-GWAS could be further increased by having larger sample sizes, higher precision with replicated phenotyping and higher marker density. It is unlikely that the power of sp-GWAS will ever rival the power of a traditional, replicated trial, plant GWAS that leverages a panel of inbred lines. There are times when a cost-benefit analysis will lead to sp-GWAS as the ideal approach, but when precision is of utmost importance a more traditional GWAS still makes sense. However, when researchers are interested in finding candidate genes in crops where association panels are not available or are time consuming to make, or when efficiency is and cost are critically important, sp-GWAS represents a potential approach to identify candidate genes for important traits. Future areas of research into the pipeline we have described herein that may be fruitful include developing a strategy for efficiently incorporating experimental checks into the field plan without introducing pollen contamination, and assessing whether or not an sp-GWAS and BSA pipeline has the potential to identify causal loci in diverse germplasm sets in addition to closed populations such as Shoepeg.

## Methods

### Plant materials and field experiments

The Shoepeg maize landrace was used as the base population for this study. Shoepeg is a southern US dent corn [90, 91]. One hundred kernels of accession PI 269743 were obtained from the National Plant Germplasm System (www.ars-grin.gov). These segregating kernels were first planted in a greenhouse where they were bulk-pollen randomly mated to generate Generation-0 seed for the experiment. In the summer of 2016, approximately 5000 seeds were bulk-planted in each of four ~ 0.1-ha plots (20,000 plants in total). Seeds were planted approximately 15 cm apart at 91 cm row spacing. Field trials were conducted in two plots in Genetics farm and two in Rollins farm near Columbia, MO. Plots were planted in isolation from other maize fields so that plants could open-pollinate without the risk of cross pollination from the other plots or other maize fields. No spatial checks were included in our experimental plots because plants were allowed to open-pollinate, and we could not allow foreign pollen to contaminate the population (see section on Bulk Segregant Analysis). In a single year, in each plot, 96 plants of the 5000 (96 × 4 = 384 out of 20,000 total plants) were chosen randomly to be genotyped and phenotyped. All 384 of the randomly chosen plants were individually measured at reproductive maturity for PH in five-centimeter increments from the ground to the collar of the flag leaf. A truncation threshold corresponding to the tallest or shortest ~ 5% of individuals in each plot was identified based on phenotypes collected from the 96 individually measured plants in each plot (Table 1, Fig. 1). Each of 5000 plants in the four plots were then phenotyped for their status above/below the truncation threshold and only ears beyond these truncation thresholds harvested. An equal number of seeds were then bulked from each location to form four new populations: Generation-1-Tall1, Generation-1-Tall2, Generation-1-Short1, and Generation-1-Short2. The four plots were chosen at random for tall- or short-plant selection.

In the summer of 2017 (year2-Generation1), the four populations were planted separately in bulks of approximately 5000 seeds again in the isolated 0.1-ha plots in the same four approximate locations in Columbia, Missouri. The process of genotyping, phenotyping, was repeated as for 2016.

### Genotyping

Leaf tissue from 96 randomly selected plants from each of the four locations for each year was collected and freeze-dried. Eight to ten leaf punches from each plant were used to extract DNA using the Qiagen DNeasy 96 plant kit, with the only modification being that samples were briefly shaken with a stainless-steel bead after addition of initial lysis buffer. DNA yield was quantified with Promega QuantiFluor on a Tecan Spark 10 M. Using 100 ng DNA and the *ApeK*I genotyping-by-sequencing (GBS) protocol [44], libraries for each of the four 96 well plates were prepared for each year. Slight modifications to the protocol included separating the 96 well into 4 pools of 24 of the adapter-ligated, pre-polymerase chain reaction (pre-PCR) pooling, and PCR amplification using ThermoFisher Phusion II master mix. Enriched library pool quantities were determined by Qubit and size distributions were checked on the Agilent Bioanalyzer high sensitivity DNA chip. All separate pools were then combined into one final pool for sequencing as there were 384 distinct barcodes to identify each sample. Barcoded adapters were designed on DeenaBIO and synthesized by IDTdna. The University of Missouri, Columbia DNA Core NEXTseq high output single end 75 bp run sequencing reads were mapped to the maize B73 reference genome version3 [AGPv3; http://ftp.maizesequence.org/ [49]] using the Tassel 5 GBS v2 pipeline [92]. This resulted in 414,361 initial SNPs with mean read depth of ~ 2.01x. Markers with minor allele frequency (MAF) < 0.05 and read count less than 40 were excluded from further analysis. SNPs were also filtered to include only diallelic loci. Imputation of missing markers was performed using Beagle version 4.1 [93]. After these filtering and imputation steps, a final dataset of 306,522 markers were used for downstream analysis.

### Phenotypic data analysis

The phenotypic data were standardized across years using a linear model where locations were treated a fixed effect with the *lm* function in R [94]. The residuals from the model were then used as the response variable for GWAS and BSA as described below. Heritability was estimated using GCTA v1.26.0 [95]. First, all genotyped SNPs were used to calculate the genomic relationship matrix (GRM) among all 768 individuals. This GRM was then used as a predictor to estimate the heritability. Principal component analysis (PCA) was performed using the R package adegenet to assess population structure [96].

### Association analysis

There are many statistical models used for association analysis, a common one being the Mixed Linear Model (MLM). Incorporating kinship and population structure in the MLM can control the false positives, but can compromise the true positives as well [97]. Fixed and Random Model Circulating Probability Unification (FarmCPU) is a model for association studies which has been shown to be effective at controlling false positive without compromising the true positives compared to other statistical models for GWAS [97]. In the FarmCPU model, to control the false positive, Multiple Loci Linear Mixed Model (MLMM) is divided into two parts: Fixed Effect Model (FEM) and Random Effect Model (REM), and these are used iteratively [97]. Model overfitting in FarmCPU is avoided by estimating kinship using associated markers in REM which is then used by FEM to test markers as covariates to control false positives and false negatives. The FarmCPU model used for GWAS in our study was done using the FarmCPU R package [97]. Generation and selection regime were incorporated in the model as covariates. Significant SNPs were defined based on a significance threshold of *P* < 0.00001. Since approximately 300,000 SNPs were tested, this threshold means that we expect fewer than three false positives across the entire set of markers. Moreover, this threshold is more conservative than others that have been used for GWAS for plant height in maize [12, 15, 31]. Genes within 150 kb of significant SNPs were manually screened for potential annotations related to PH. Annotations were downloaded from Ensembl (http://ensembl.gramene.org/Zea_mays/Info/Index) and the MaizeGDB database (http://www.maizegdb.org/gbrowse).

### Bulk segregant analysis

A modified form of bulk segregant analysis (BSA) was performed by evaluating the 384 plants observed in Generation-1.While the original method of RW Michelmore*,* et al. [39] used bi-parental populations in their analysis, we used a segregating population as a base which is also akin to one-generation selection experiment. BSA is not an inherent necessity of sp-GWAS, but we believe that combination of BSA with GWAS provided a strong corroboration of the candidate that we identify, and these approaches complement each other well in one pipeline. The 384 randomly chosen plants genotyped in Generation-0 provided an estimate of the base allele frequencies. Then, the 384 randomly chosen plants genotyped in Generation-1 provided an estimate of the allele frequencies of the 5% tallest and shortest plants from Generation-0 for BSA. Markers were first filtered for > 0.05 MAF and read count greater than 40. After filtering, 243,303 SNPs were used for further analysis. The frequency of the reference allele at each site was estimated using the “sm” R-script from Haase et al. [68]. Significance for each locus was computed by using a two-sided Z test. To identify the significant SNP, first the significant region was identified that included all the SNPs with -log10(*p*-value) over the outlier threshold of 0.5% [98]. Then a 15-SNP sliding window was applied to smooth results [68].

## Additional files

Additional file 1: Estimation of heritability using the breeder’s equation: R = h^2^S, where S is the difference between the population average and average of selected individual from Gen-0, R is the difference between the population average from Gen-0 and the average of the individuals in Gen-1 and h^2^ is the heritability. (DOCX 12 kb)

Additional file 2: Population structure based on the principal component analysis (PCA) for 768 Shoepeg plants used in the association analysis. Panel (a) shows the relationship of PC1 vs PC2 for the two generations and panel (b) shows the relationship of PC1 vs PC2 using selection regime. (TIFF 362 kb)

Additional file 3: Quantile-quantile (Q-Q) plots for Plant Height GWAS using FarmCPU. The red line is the 1:1 identity line. (PNG 73 kb)

## Data Availability

All the data and statistics about the present study has been included in the current manuscript in the form of figure and tables. Raw data are publicly available at figshare; https://figshare.com/s/4a9620c8752355a04e2a. Our analysis code is available publicly on github; https://github.com/abi01/sp-GWAS.

## Abbreviations

BR: Brassinosteroids
BSA: Bulk Segregant Analysis
FarmCPU: Fixed and Random Model Circulating Probability Unification
GA: Gibberellin
GBS: Genotype by Sequencing
GRM: Genomic Relationship Matrix
GWAS: Genome Wide Association Study
MAF: Minor Allele Frequency
PCA: Principle Component Analysis
PH: Plant Height
QTL: Quantitative Trait Loci
QTN: Quantitative Trait Nucelotide
SNPs: Single Nucleotide Polymorphism
sp-GWAS: Single Plant GWAS
