# Supplementary material for: Single-plant GWAS coupled with bulk segregant analysis allows rapid identification and corroboration of plant-height candidate SNPs
Source: BMC Plant Biol. 2019 Oct 8;19:412. doi: 10.1186/s12870-019-2000-y (PMC6781408; doi:10.1186/s12870-019-2000-y)
Supplement: Supplementary file 1 — Estimation of heritability using the breeder’s equation: R = h2S, where S is the difference between the population average and average of selected individual from Gen-0, R is the difference between the population average from Gen-0 and the average of the individuals in Gen-1 and h2 is the heritability. (DOCX 12 kb) [file 12870_2019_2000_MOESM1_ESM.docx]

| Location | Gen-0 (Average) | Average of Selected | Gen-1 (Average) | S | R | h^2^ |
| --- | --- | --- | --- | --- | --- | --- |
| Tall1 | 184.78 | 215 | 201.5 | 30.22 | 16.72 | 0.55 |
| Tall2 | 192.5 | 220 | 200.5 | 27.5 | 8 | 0.29 |
| Short1 | 175.58 | 140 | 168.125 | -35.58 | -7.455 | 0.21 |
| Short2 | 176.83 | 152 | 172.05 | -24.83 | -4.78 | 0.19 |
