# Supplementary figures and images for: Single-plant GWAS coupled with bulk segregant analysis allows rapid identification and corroboration of plant-height candidate SNPs
Source: BMC Plant Biol. 2019 Oct 8;19:412. doi: 10.1186/s12870-019-2000-y (PMC6781408; doi:10.1186/s12870-019-2000-y)

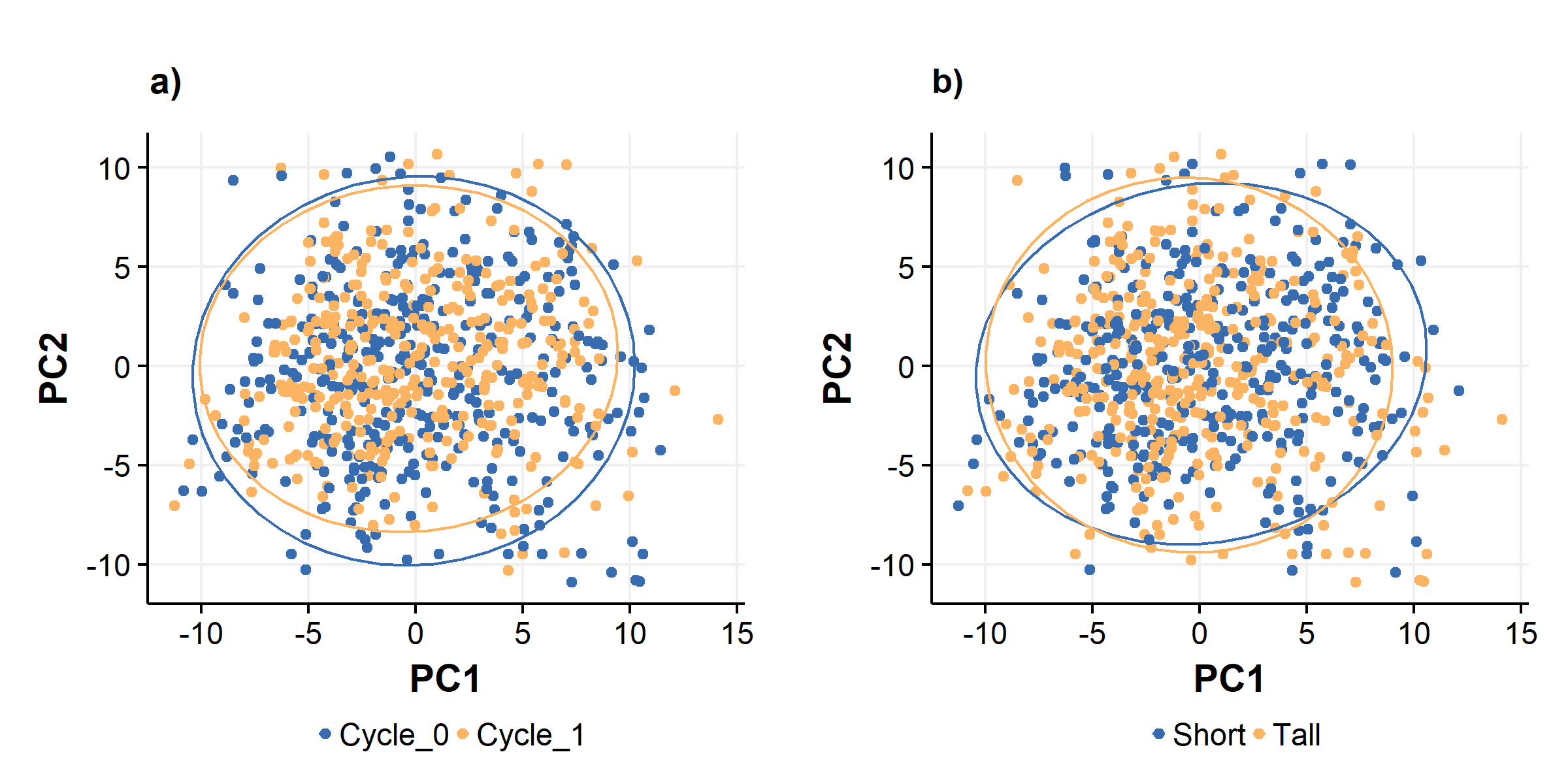

Supplement: Supplementary file 2 — Population structure based on the principal component analysis (PCA) for 768 Shoepeg plants used in the association analysis. Panel (a) shows the relationship of PC1 vs PC2 for the two generations and panel (b) shows the relationship of PC1 vs PC2 using selection regime. (TIFF 362 kb) [file 12870_2019_2000_MOESM2_ESM.tiff]

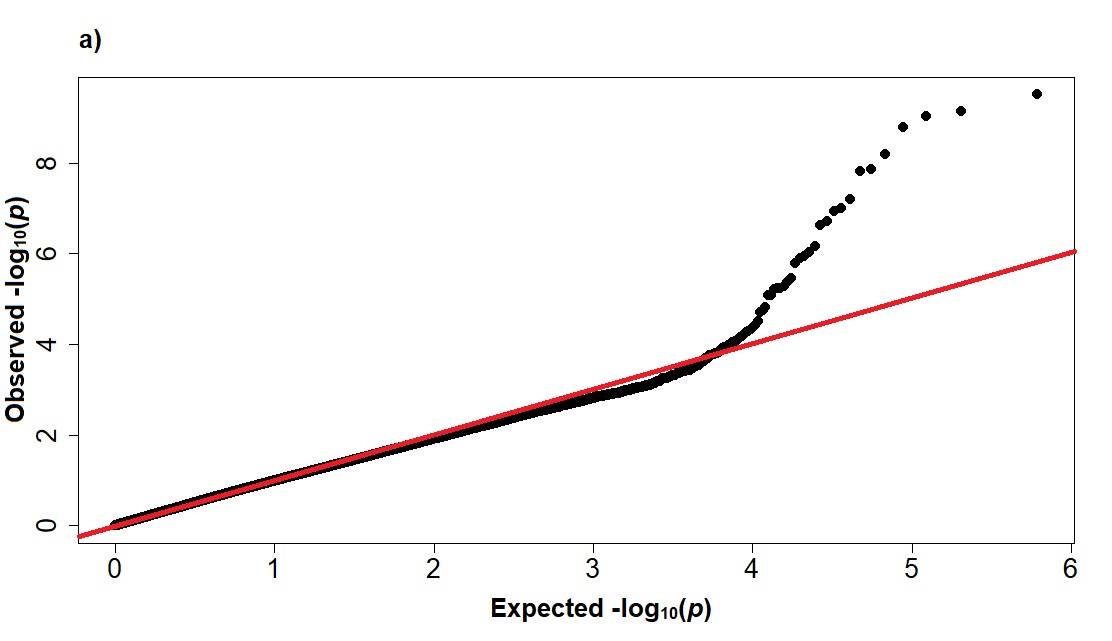

Supplement: Supplementary file 3 — Quantile-quantile (Q-Q) plots for Plant Height GWAS using FarmCPU. The red line is the 1:1 identity line. (PNG 73 kb) [file 12870_2019_2000_MOESM3_ESM.png]
